# Understanding Breastfeeding Practices in Tennessee: Goals, Duration, and Reasons for Discontinuation

**DOI:** 10.13023/jah.0704.01

**Published:** 2025-12-01

**Authors:** Amir Alakaam, Chia-Lin Chang, Hannah Jeffery

**Affiliations:** University of Tennessee at Chattanooga; University of North Dakota; University of Tennessee at Chattanooga

**Keywords:** Appalachia, breastfeeding, breast milk, infant formula, nutrition, childhood

## Abstract

**Introduction:**

Breastfeeding is widely recognized as the optimal source of infant nutrition, offering many health benefits.

**Purpose:**

The purpose of this study was to examine goals, exclusivity, and duration of breastfeeding and explore factors that influence breastfeeding practices among individuals in Tennessee (TN).

**Methods:**

A cross-sectional study was conducted among individuals (N = 1,249). Participants were recruited and data were collected from June – September 2021. The data were entered and analyzed from September 2022 – March 2023. Descriptive statistics, chi-square test, and ANOVA were used to analyze the data and identify associations between variables.

**Results:**

About 32% of the participants did not meet their goal to exclusively breastfeed for six months. Most participants reported that they chose to breastfeed due to benefits for the child (78.4%) and woman (62.1%). About 44% of the participants indicated that their healthcare provider promoted milk formula, and 41% did not feel comfortable breastfeeding in public. More than half of the participants reported not receiving education on the benefits of breastfeeding from their healthcare provider. Findings showed a significant relationship between discontinuing exclusive breastfeeding early and not receiving support from hospital maternity staff (X2 (1, N = 403) = 7.7, *p* < 0.05) and not feeling comfortable breastfeeding in public (X2 (1, N = 372) = 15.5, *p* < 0.001).

**Implications:**

Enhanced education and support from healthcare providers, along with efforts to normalize breastfeeding in public, are essential to improve breastfeeding outcomes and support individuals in achieving their breastfeeding goals.

## INTRODUCTION

Adequate nutrition during infancy is crucial for optimal growth, development, and health of children. The current recommendations from the American Academy of Pediatrics (AAP) advise exclusive breastfeeding for six months and introducing complementary foods while continually breastfeeding until the infant is at least 1 year old.^1^ Exclusive breastfeeding can be defined as giving only breast milk, with the exclusion of mixing it with any food or liquid, in the first six months of life. Breastfeeding is widely recognized as the optimal source of infant nutrition, offering many health benefits. It enhances the infant’s immune system, reducing the risk of infections such as respiratory illnesses, diarrhea, and ear infections. Additionally, breastfeeding supports cognitive development and emotional well-being, fostering stronger maternal-infant bonding. Moreover, it lowers the likelihood of chronic conditions like asthma, obesity, and type 2 diabetes later in life.^1^–^4^

Across the United States (U.S.), 83.2% of the population initiated breastfeeding, while only 55.8% continued breastfeeding up to six months, 35.9% up to one year, and only 24.9% of infants were exclusively breastfed for six months.^3^ Specifically, in Tennessee (TN), 78.8% of women started breastfeeding, but only 53.2% continued for six months, and 31.5% for one year, all of which fall short of the Healthy People 2030 Objectives.^3^,^5^ Additionally, the COVID-19 pandemic has reshaped breastfeeding by reducing access to healthcare services and increasing social isolation.^6^–^7^ In TN, limited healthcare infrastructure, socioeconomic challenges, and poor broadband access made it harder for mothers to receive lactation support, further widening existing disparities in breastfeeding practices.^8^–^9^ Understanding the impact of these changes on breastfeeding practices is essential for developing effective interventions and policies to support breastfeeding women.

Fifty-two of TN’s 95 counties are located in the Appalachian region, an area characterized by well-documented health disparities, including challenges in maternal and child health.^8^–^9^ Factors such as limited access to healthcare resources and socioeconomic constraints may further affect breastfeeding initiation and continuation in these communities.^9^ Despite these challenges, research specifically examining breastfeeding practices within TN, and particularly in Appalachian counties, remains limited. Moreover, while existing statewide data highlight the importance of understanding breastfeeding outcomes, there is a notable lack of studies examining breastfeeding practices and barriers in TN

This study aims to examine breastfeeding goals, exclusivity, and duration among individuals in TN, and to explore the factors that influence breastfeeding practices. The study further seeks to identify associations between breastfeeding behaviors and relevant sociodemographic and contextual variables, including reasons for breastfeeding initiation and discontinuation.

## METHODS

This is a cross-sectional study that used a self-administered survey. This study was in accord with prevailing ethical principles and approved by the Institutional Review Board at the University of Tennessee at Chattanooga. Participants who were included in this study are female, 18 years and over, living in TN, fluent in the English language, and have given birth in the past three years. Participants were recruited from June – September 2021 by posting advertisements on Facebook, a widely used social media platform, to maximize outreach to potential participants across TN. In addition, flyers containing study information and the survey link were distributed and posted in maternity and pediatric health facilities, including birthing units, throughout various regions of the state. The collective recruitment strategies were designed to enroll participants representing a wide range of characteristics, ensuring broad community representation across TN.

We utilized an online survey generated through QuestionPro.^10^ A QuestionPro QR code and website address were included in each flyer and advertisement that enabled the participant to complete the online survey.^10^ The survey was offered in English and took 10–15 minutes to complete.

The survey instrument contained four sections. The first section consisted of inclusion criteria and questions related to goal, initiation, duration, and practices of breastfeeding. The second and third sections asked about factors that influence breastfeeding including facilitators and barriers to breastfeeding based on the participants’ own experience with their most recent birth. In this section, we also asked specific questions related to breastfeeding during the COVID-19 pandemic. In the fourth section, participants were asked to provide sociodemographic information including age, race, marital status, education level, household income, and employment status. The questionnaire also included an open-ended question that asked the participant to provide any suggestions to increase breastfeeding support. Questions were adapted from previous studies and literature.^11^–^14^ A pilot test among a sample of 10 participants was conducted to confirm instrument reliability and consistency. Pilot participants met the same eligibility criteria as the main study sample ensuring comparability in key characteristics.

At the beginning of the survey, a consent form and disclosure information of the study were shown, and participation was completely voluntary. Participants started taking the survey only if they consented to do so. All the information obtained was anonymous. Participants’ responses were not linked to the consent forms. The participant’s name, and any other personal identifiers were not collected in any form. The first 100 participants who completed the questionnaire received a $20 gift card through an external link that was not tied to survey responses. For participants beyond the initial 100, completion of the questionnaire provided entry into a drawing for five $20 gift cards.

The data were entered and analyzed from September 2022 – March 2023 using IBM SPSS Statistics version 28.^15^ Descriptive statistics were indicated for the following variables: breastfeeding goal, breastfeeding duration, breastfeeding exclusivity, reason to breastfeed, reason to discontinue breastfeeding, factors influencing breastfeeding, and other variables. Breastfeeding duration, age at infant formula introduction, and age at introduction of foods or liquids were treated as continuous variables and summarized using medians and interquartile ranges (IQRs) due to skewed distributions. Sociodemographic variables (e.g., age group, race, marital status, education level, household income, employment status) and breastfeeding-related factors (e.g., initiation timing, goals, reasons for breastfeeding, reasons to discontinue breastfeeding) were treated as categorical variables and summarized using frequencies and percentages. Pearson correlation coefficients were calculated to assess the strength and direction of relationships between continuous variables (e.g., age of the child at discontinuation of exclusive breastfeeding and age at introduction of infant formula). One-way analysis of variance (ANOVA) was used to compare mean breastfeeding duration across categorical groups; for statistically significant results, means and standard deviations were reported alongside F statistics and p-values. Chi-square tests of independence were conducted to evaluate associations between categorical variables (e.g., reasons for breastfeeding and exclusive breastfeeding status).

All tests were two-tailed, with a significance threshold of *p* < 0.05. For inferential analyses, complete-case analysis was applied, restricting the sample to participants with non-missing data on all variables included in the model. Descriptive statistics were calculated using all available data for each variable; therefore, denominators vary across tables and results.

## RESULTS

### Participant Characteristics

A total of 1,381 individuals participated in this study. Due to missing data in some variables, the number of participants included in the main analysis varied by variable. For the primary analysis, 1,249 participants had complete data on all key variables and were included. The majority of participants were aged 25–34 (64.2%), white (73.0%), married (87.7%), have an income of $40,000 or above (62.3%), and work full-time (51.5%). More than half of the participants reported having only one child (56.8%), the median age for each child was 1 year, and vaginal birth was the main mode of delivery (82.4%). Participants’ characteristics are in Table 1.

### Breastfeeding Goal, Initiation, Exclusivity, Duration

Participants were asked about their goals in relation to breastfeeding their child. Almost all the participants (n=1214, 97.2%) reported planning to breastfeed their child. About 32% (n=382) indicated having a goal of three months of exclusive breastfeeding, and about 37% (n=382) indicated having a goal of six months of exclusive breastfeeding. More than half (n= 667, 55.7%) had the goal to breastfeed for 12 months, and only 14.3% (n=171) indicated having a goal to breastfeed for 24 months.

About 90% (n=304) of participants achieved their goal of three months of exclusive breastfeeding. However, 32.1% (n= 128) of the participants did not meet their goal to exclusively breastfeed for six months, and 63.8% (n=354) did not accomplish their 12 months goal of breastfeeding. Only 26.9% (n=105) of participants exclusively breastfed their child for less than six months, and 18.7% (n=73) of participants exclusively breastfed their child for six months.

About 40% (n=486) of the participants indicated feeding their child infant formula. The median age of the child when their mother introduced infant formula was three months (IQR: 4 – 240 days). The median age of the breastfed child when their mother gave them foods or liquids was six months (IQR: 150 – 360 days). Among the participants who completely discontinued breastfeeding (n = 356), 27.7% (n = 99) of women discontinued breastfeeding when their child reached six months, 38.4% (n = 137) when their child was between six and 12 months, and 29.1% (n = 104) when their child was between 12 and 24 months. About 5% (n=19) of women discontinued breastfeeding their child after 24 months.

The majority of the participants initiated breastfeeding before being discharged from the hospital (n=1020, 85.9%), and more than half stated they breastfeed their child soon after birth (n=696, 58.6%). About 29% (n=341) of participants reported breastfeeding their child between one to six hours, 3.5% (n=42) more than six hours, and 7% (n=83) over 24 hours after birth. A post hoc analysis indicated that the participants who breastfed their child between one to six hours after birth had a longer extended period of exclusive breastfeeding than any other group (within one hour, *p* <0.001; over 24 hours, *p* <0.001). There was no difference found between breastfeeding after more than six hours or over 24 hours after birth.

A Pearson correlation was performed to analyze the strengths and direction of the relationship between the discontinuance of exclusive breastfeeding, the introduction of infant formula, and supplemental foods or liquids. Findings showed that there was only a moderate, positive correlation between the age of the child when exclusive breastfeeding was discontinued and the age of children when the infant formula was introduced (r =.66, *p* <0.001), or the age of children when they first received foods or liquids other than breast milk (r =.59, *p* <0.001).

ANOVA was used to analyze the association between breastfeeding duration and participant characteristics. Findings indicated race (F 7,389 = 6.33, *p* <0.001) and age (F 7,389= 2.61, *p* <0.05) influenced the mean exclusive breastfeeding duration; however, a post hoc analysis cannot be done because some groups had few cases. Findings also showed a significant relationship between marital status and breastfeeding duration (F 4,319 = 4.58, *p* =0.001), Fisher’s Least Significant Difference test for multiple comparisons found that the married participants had a significantly higher breastfeeding duration when compared to participants who reported being separated (*p* < 0.001). There was no significant difference between other variables and duration of breastfeeding.

### Reasons for Breastfeeding

The majority of participants reported that they chose to breastfeed because of breastfeeding benefits to the child (n=907, 78.4%) followed by breastfeeding benefits to the woman (n=719, 62.1%) and convenience of breastfeeding (n=613, 53.0%). Some participants reported that the reason they chose to breastfeed was because breastfeeding would help them lose weight after pregnancy (n=445, 38.5%) and breastfeeding is a tradition and normal practice in their culture (n=344, 29.7%). Additionally, participants reported they believed that breastfeeding would protect their child from contracting COVID-19 (n=341, 29.5%). Participants who answered other reasons in the open-ended question (n=99) stated they chose to breastfeed because formula is more expensive (n= 48, 48.5%), and many reported they wanted to bond with their child through breastfeeding (n=26, 26.2%).

A chi-square test indicated a significant association between reporting “benefits of breastfeeding” as a reason to breastfeed and meeting the six months exclusive breastfeeding (X2 (1, N = 392) = 11.0, *p* < 0.001). The majority of participants (n=240, 88.2%) who followed the six months recommendation were aware of the benefits of breastfeeding for both mother and child. A chi-square test of independence showed that there was no significant association between reason to breastfeed and other variables.

### Reasons to Discontinue Breastfeeding

Insufficient breast milk was the top reason to discontinue breastfeeding (12.1%) followed by “could not breastfeed in public” (10.4%), and “sore, cracked, or bleeding nipples” (9.9%). See reasons for discontinuance of breastfeeding as perceived by the participant in Table 2.

Participants were also asked if they discontinued breastfeeding for a specific reason related to the COVID-19 pandemic. About 5% (n=54) of participants reported that they had discontinued breastfeeding due to quarantine, and 3.6% (n=42) discontinued breastfeeding due to testing positive for to COVID-19. Only 4.2% (n=49) reported discontinuing breastfeeding due to being scared of transmitting the virus to their child, and 4.3% (n=50) because they received the COVID-19 vaccine.

### Factors Influencing Breastfeeding

Participants were asked about factors that influence breastfeeding as facilitators or barriers. The majority of participants indicated receiving support from their spouse (87.6%), family members (81%), or friends (65.1%). About 44% of the participants indicated that their obstetrician or gynecologist promoted infant formula, and 41% did not feel comfortable breastfeeding in public. More than half of the participants reported not receiving education on the benefits of breastfeeding from their child’s pediatrician during their regular well-baby visit (51.6 %) nor from their obstetrician or gynecologist (56.2%). See Table 3 for a list of factors influencing breastfeeding based on participants’ experience with their most recent birth.

## DISCUSSION

In this study, breastfeeding goals, exclusivity, and duration were examined, as well as the factors that influence breastfeeding among participants in TN. The findings revealed that while many of the participants initiated breastfeeding, many were met with barriers that caused them to discontinue breastfeeding. Only a few of the participants met their goal for breastfeeding and followed the recommended guidelines to exclusively breastfeed for six months.^1^ Many participants experienced barriers to breastfeeding, causing them to not meet the recommended guidelines. Previous research in the U.S. has identified similar barriers related to breastfeeding, such as lack of support from family, peers, and hospital staff which is consistent with our findings.^16^

It was interesting to find that most of the participants did not receive appropriate education on benefits of breastfeeding or support related to breastfeeding from their obstetrician, gynecologist, pediatrician, or hospital staff. The authors recommend that health facilities provide training on standard practices related to breastfeeding to health providers and staff to ensure accurate support of breastfeeding.

One way to increase breastfeeding support and overcome barriers related to breastfeeding within a health facility and among staff is by following the Baby-Friendly Hospital Initiative (BFHI) and the Ten Steps to Successful Breastfeeding guidelines.^17^–^19^ While full BFHI designation may be challenging for facilities in resource-limited settings, such as many rural and Appalachian communities, where hospital closures and staffing shortages have been exacerbated by the COVID-19 pandemic, key BFHI principles can still be adapted and implemented in a variety of care settings. Evidence suggests that even partial adoption of BFHI practices, such as early skin-to-skin contact, rooming-in, and provider counseling on exclusive breastfeeding, can improve breastfeeding initiation and duration. 18–20

In addition to facility-based strategies, these findings highlight the importance of community and public level support. A substantial number of participants reported breastfeeding discomfort in public, indicating a need for interventions to normalize breastfeeding outside the home. Public breastfeeding education campaigns and the creation of breastfeeding-friendly public spaces could help alleviate this barrier.^21^–^22^

The findings showed that receiving assistance with breastfeeding from maternity providers led to a longer duration of breastfeeding among participants. It also showed that some obstetrician-gynecologists promoted infant formula among many participants. Although infant formula should only be provided if there is a medical indication, it had been reported that about 30% of infant formula supplementation was not medically indicated in the U.S.^23^–^24^ Maternity and pediatric health providers should be mindful of providing encouragement to using infant formula if there is no medical indication. Studies have shown that maternity staff have a big influence on an individual’s decision to breastfeed. The results of these studies align with our findings.^25^–^27^

Although introducing infant formula or giving supplemental foods or liquids to children was the origin of discontinuing exclusive breastfeeding, our research found that there was only a moderate correlation between the age of children when exclusive breastfeeding was interrupted and the age of children when infant formula was introduced or the age of children when they first received foods or liquids other than breast milk. The moderate correlation between the child’s age when discontinuing exclusive breastfeeding and statement of exclusively breastfeeding may suggest that there are participants who do not know that introducing infant formula or food and other liquids to their child is considered breaking exclusive breastfeeding. The definition of exclusive breastfeeding was given in the survey clearly as “the baby receives only breast milk (no other liquids or formula).” However, there was reliance on misinformation related to exclusive breastfeeding and what was considered as breaking exclusive breastfeeding. Health agencies should provide clear information about breastfeeding exclusivity so it will be easier for individuals to understand and follow the guidelines. Data related to introducing infant formula among participants is presented in other report.^28^

This study had limitations. Data collection relied on self-reporting, which may be subject to recall bias, especially given the partially retrospective nature of the survey. Future studies should consider longitudinal designs or distinguish between individuals currently breastfeeding and those who have completed breastfeeding to better capture changing outcomes. Although the adapted and modified instrument used here was reviewed by a committee of experts for validity and survey questions were pilot tested for reliability, the instrument may exhibit some limitations. Collected data came from only one state in the U.S.; researchers should be mindful of the generalizability of this study. In this study, data were limited to those who gave birth within the last three years. In future studies, researchers may consider separating individuals who are currently breastfeeding and individuals who have completed breastfeeding. This study also provided data related to the impact of the COVID-19 pandemic on breastfeeding. More detailed findings are reported in another publication.^29^

## IMPLICATIONS

Despite substantial evidence supporting breastfeeding, these findings indicate that many individuals in TN initiate breastfeeding but discontinue it earlier than recommended, often introducing formula or complementary foods prematurely. These trends reflect ongoing challenges in breastfeeding support and education, particularly in Appalachian regions where health disparities persist.^30^ Given the multifactorial nature of breastfeeding behaviors, it is essential for public health agencies, healthcare providers, and community organizations to prioritize comprehensive breastfeeding support within maternal and child health initiatives. Of particular concern is the frequent and medically unnecessary use of infant formula which underscores the urgent need for enhanced provider training and institutional policies that encourage breastfeeding.

The findings also reveal a significant knowledge gap regarding the definition and importance of exclusive breastfeeding. This highlights the need for clear, consistent, and accessible education strategies to improve understanding and confidence in breastfeeding practices. To address these challenges and improve breastfeeding outcomes, the adoption and expansion of evidence-based programs such as the Baby-Friendly Hospital Initiative (BFHI) in TN’s healthcare facilities are recommended.^18^ The BFHI promotes hospital policies and practices that support breastfeeding initiation and continuation, helping align local practices with national health guidelines.^18^–^19^ Additionally, efforts to normalize breastfeeding in public through education and supportive environments are needed to reduce discomfort and promote breastfeeding confidence in public.^21^–^22^

Overall, these efforts are critical to narrowing the gap between recommended breastfeeding guidelines and actual practices, thereby promoting healthier outcomes for families throughout TN and the U.S.

**SUMMARY BOX**

**What is already known about this topic?**

Previous studies have identified various barriers to breastfeeding in the U.S., including lack of support from family, healthcare providers, and hospital staff. Research has also shown that the implementation of initiatives such as the Baby-Friendly Hospital Initiative (BFHI) can improve provider practices and breastfeeding outcomes. However, gaps remain in consistent education and support for breastfeeding among healthcare providers and in public understanding of breastfeeding guidelines.

**What is added by this report?**

The findings contribute to the literature by providing updated, region-specific insights into breastfeeding practices in a U.S. Southern state, which is often underrepresented in national breastfeeding research. Our analysis adds value by identifying key patterns and barriers to continued breastfeeding, with implications for maternal and child health practices and hospital setting

**What are the implications for future research?**

Future research should explore strategies to improve provider training on breastfeeding support and assess the effectiveness of standardized interventions like BFHI in various clinical settings. Studies should also evaluate public understanding of exclusive breastfeeding definitions and guidelines, aiming to close the knowledge gap and promote adherence to evidence-based practices.

## Figures and Tables

**Table 1:** Participant characteristics (N=1,249)

Characteristic		n	%*
**Age**	18–29	592	51.2
	30–39	507	43.8
	40 and above	58	5.0
**Race**	White	845	73.0
	Black or African American	166	14.3
	American Indian or Alaska Native	38	3.3
	Asian	30	2.6
	Native Hawaiian or other Pacific Islander	6	0.5
	Hispanic	22	1.9
	Other	4	0.3
	Two or more races	46	4.0
**Marital status**	Single	39	3.4
	Married	1015	87.7
	Separated or divorced	42	3.6
	Cohabitating (living with significant other but not married)	61	5.3
**Highest level of education**	Less than a high school diploma	106	9.2
	High school diploma	326	28.2
	Associate’s degree or vocational degree	211	18.2
	Bachelor’s degree	379	32.8
	Master’s degree	109	9.4
	Doctoral degree	26	2.2
**Household income**	Below 10,000	15	1.3
	10,000 to 39,999	411	36.4
	40,000 to 79,999	425	37.7
	80,000 or above	277	24.6
**Employment status**	Employed full-time	596	51.5
	Employed part-time	277	23.9
	Unemployed	284	24.5

NOTES:

* Counts and percentages in this table are based on available responses for each variable; sample sizes for descriptive statistics may differ from the analytic sample size

**Table 2:** Reason to discontinue breastfeeding as perceived by participant (N=1,249)

Reason	n*	%
Insufficient milk supply	140	12.1
Could not breastfeed in public	120	10.4
Sore, cracked, or bleeding nipples	115	9.9
Workplace did not support breastfeeding woman	97	8.4
Breastfeeding was painful	91	7.9
Change of body weight or shape	86	7.4
Lack of support from health professional	80	6.9
Changed sexual feelings, attractiveness, or drive	56	4.8
Lack of support from child pediatrician	51	4.4
Leaked milk too much	49	4.2
Tired	45	3.9
Lack of support from family	44	3.8
Child not satisfied with breast milk	43	3.7
Impact of sexual relationship with partner	41	3.5
Became pregnant	41	3.5
Did not like breastfeeding	24	2.1
Lack of support from spouse	22	1.9
Wanted to exercise	8	0.7
Wanted to smoke or drink alcohol	6	0.5

NOTES:

* Number and percentage were calculated based on number of individuals who answered yes to specific question.

**Table 3:** Factors influencing breastfeeding based on participants’ experience with most recent birth (N=1,249)

Factor	n*	%
Received spouse’s support to breastfeed	1013	87.6
Received hospital staff support in starting breastfeeding	929	86.1
Received support from family members to breastfeed	937	81.0
Prepared to breastfeed prior to giving birth	827	80.3
Birthing facility supported breastfeeding	755	78.2
Aware of benefits of breastfeeding over formula	861	74.4
Aware of financial savings of breastfeeding over formula	817	70.6
Once a breastfed baby	782	67.6
Utilized breastfeeding support services while in the birthing hospital	656	66.1
Received friends’ support to breastfeed	753	65.1
Felt comfortable breastfeed in public	576	59.3
Received education from child’s pediatrician on the benefits of breastfeeding during regular well-baby visit	560	48.4
Participated in pre-birth, childbirth, or pregnancy education classes	534	46.2
Received education of breastfeeding from Obstetrician-gynecologist	507	43
Received education of breastfeeding from pre-birth, childbirth, or pregnancy education classes	488	42.2
Felt like are not producing enough milk	545	53.1
Obstetrician-gynecologist promoted infant formula	464	43.7
Child had medical reasons interfered with breastfeeding	317	31.4
Had medical reasons interfered with breastfeeding	229	22.5

NOTES:

* Number and percentage were calculated based on number of individuals who answered yes to specific question
